# Histological Justification for the Need of Radiofrequency Ablation of Pulmonary Arteries in Patients with High-Grade Secondary Pulmonary Hypertension

**DOI:** 10.17691/stm2021.13.6.06

**Published:** 2021-12-28

**Authors:** N.A. Trofimov, A.L. Rodionov, D.V. Egorov, T.V. Surkova, A.V. Nikolsky

**Affiliations:** Leading Researcher; Chuvash State University named after I.N. Ulyanov, 15 Moskovsky Prospect, Cheboksary, Chuvash Republic, 428015, Russia; Researcher; Chuvash State University named after I.N. Ulyanov, 15 Moskovsky Prospect, Cheboksary, Chuvash Republic, 428015, Russia; Junior Researcher; Chuvash State University named after I.N. Ulyanov, 15 Moskovsky Prospect, Cheboksary, Chuvash Republic, 428015, Russia; Laboratory Assistant; Chuvash State University named after I.N. Ulyanov, 15 Moskovsky Prospect, Cheboksary, Chuvash Republic, 428015, Russia; Researcher; Chuvash State University named after I.N. Ulyanov, 15 Moskovsky Prospect, Cheboksary, Chuvash Republic, 428015, Russia

**Keywords:** secondary pulmonary hypertension, radiofrequency ablation, denervation of the pulmonary arteries

## Abstract

**The aim of the study** was to perform a histological assessment of the effectiveness of radiofrequency exposure for circular denervation of the pulmonary artery in patients with secondary high pulmonary hypertension.

**Materials and Methods:**

The study was carried out on the autopsy material derived from non-operated patients. Three groups were formed. The experimental group included the material (207 histological samples) from the patients with chronic high pulmonary hypertension arising on the background of mitral heart disease. The samples of this group were exposed to circular radiofrequency ablation. In the comparison group, we used autopsy material (24 samples) obtained from the patients with high pulmonary hypertension. The control group included material (35 samples) from the patients without pulmonary hypertension who died from causes not associated with cardiovascular diseases. The samples of the comparison and control groups were not exposed to radiofrequency.

Visual evaluation of the damage to the vascular wall was performed after hematoxylin and eosin staining, according to Van Gieson. Damage to the nerve plexuses was evaluated after their impregnation by silver salts. To assess the degree of damage to the vascular wall on the stained sections, a scoring method of semi-quantitative analysis of the observed pathological processes (fibrinoid necrosis, metachromasia, karyorrhexis, karyolysis, fibrinoid and mucoid swelling, lipid presence) was used. Silver salt impregnation allowed visualizing damage to the reticular fibers, trunks and endings of peripheral nerve fibers.

**Results:**

The mean optical density of the ablation group was statistically significantly lower than in the comparison and control groups (p<0.001). The mean specific area of tissue dissociation was higher in the “marginal zones” of the ablated sections, under pronounced mechanical compression in these areas. The difference in the mean areas of the argentophilic samples of the ablation and comparison and control groups was expressed in a lower percentage of argentophilic fibrous structures (p<0.05). At the same time, the highest concentration of argentophilic structures was observed in the comparison group, which points to a bigger content of nerve fiber structures in the patients with high pulmonary hypertension.

**Conclusion:**

The results of the histological study demonstrated the feasibility of radiofrequency ablation of the pulmonary arteries in patients with high-grade secondary pulmonary hypertension. Radiofrequency denervation leads to the destruction of the sympathetic ganglia in the adventitial layer of the pulmonary arteries, which are responsible for the spasm of the precapillary bed of the pulmonary circulation, which promotes vasodilation, an increase in the vascular bed, and, as a result, a reduction in pulmonary hypertension.

## Introduction

Diseases of the cardiovascular system remain among the most common human diseases. In particular, the prevalence of mitral valve disease reaches 8%, and numerous complications require an individual surgical approach [1, 2].

Disease of the mitral valve, along with its natural course, results, primarily, in dilatation of the left atrium, congestion in the pulmonary circulation, secondary pulmonary hypertension (PH), impairment of the electrophysiological features of the myocardium with the appearance of pathological reentrant circles, and the formation of atrial fibrillation (AF) as well as the progression of comorbid pathology [3, 4].

Atrial fibrillation is the most frequent arrhythmological complication in patients with valvular heart disease observed in 30–50% of cases. Concomitant AF reduces the effectiveness of surgery and affects the postoperative course in this category of patients: it significantly increases the risk of thromboembolic complications, promotes the progression of heart failure and, as a result, increases overall mortality [5–7].

Correction of valvular disorders is necessary for the successful treatment of AF [1]. However, in most cases, this is not enough: surgical treatment of a mitral defect in patients with preoperative AF provides restoration of sinus rhythm only in 8.5–20.0% of cases, thereby, additional surgical intervention is required [5, 8].

High PH levels in patients with valvular heart disease reduce the effectiveness of surgical treatment, the rate of postoperative remodeling of cardiac cavities, and also affect the maintenance of sinus rhythm in patients with AF after the Maze IV procedure [6, 9, 10].

Pulmonary hypertension is conventionally considered to be primary and secondary. Primary PH is mediated by 2q33 chromosome mutation which is responsible for the growth and proliferation of endothelial cells. Secondary PH develops in patients with systemic diseases, lesions of the left heart, metabolic disorders, pulmonary thromboembolism, and pathology of the respiratory system [11–13].

The current classification involves five main clinical phenotypes of PH:

pulmonary arterial hypertension;

pulmonary hypertension associated with left heart diseases;

pulmonary hypertension associated with pathology of the respiratory system and/or hypoxemia;

chronic thromboembolic pulmonary hypertension and other types of pulmonary artery (PA) obstruction;

pulmonary hypertension with unclear and/or multiple mechanisms [14].

Conservative methods of treatment of high-grade PH do not have a significant effect on the course of the disease in all patients and are associated with high financial costs [15].

In 2013, Chen et al. [16] for the first time proposed a surgical method for correcting high PH, which consisted of circular endovascular catheter ablation of the PA trunk and orifices of the right and left PA. The immediate results of the treatment were encouraging and contributed to a significant reduction in PH.

Later on, surgical treatment for PH in patients with mitral valve pathology during cardiac surgery with cardiopulmonary bypass was proposed. The technique of using a monopolar electrode when performing radiofrequency ablation of the anterior wall of the PA trunk and orifices was described [17]. A method of applying a bipolar ablation clamp to carry out circular pulmonary denervation was proposed [18].

In spite of the active search for surgical methods for correcting PH, no conventional guidelines for the treatment of this disease have been worked out, and no histological evidence has been presented for the feasibility of radiofrequency exposure to the PA walls during cardiac surgery.

**The aim of the study** was to perform a histological evaluation of the effectiveness of radiofrequency exposure in pulmonary artery denervation in patients with secondary high pulmonary hypertension.

## Materials and Methods

**Figure 1.**
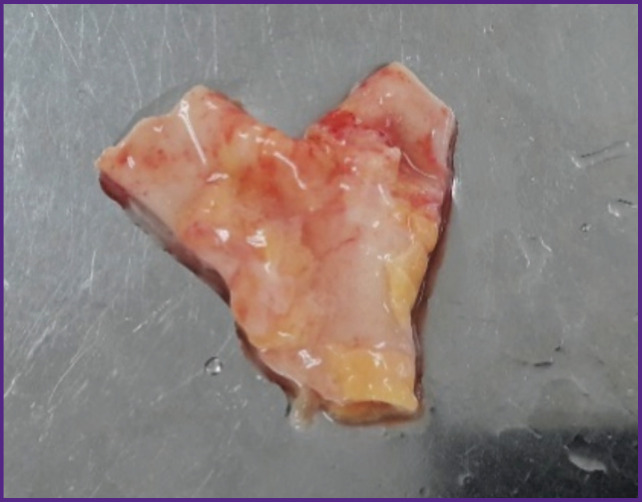
Sample of the pulmonary artery with branches

The study was carried out on the autopsy material of the pulmonary trunk with the right and left PA branches derived from non-operated patients (n=9) within 3 h from the moment of biological death (Figure 1).

Three groups of 3 patients each were formed. Group 1, experimental, included histological tissues from the patients with chronic high PH, which arose due to mitral heart disease. The samples in this group (207 histological samples) were exposed to circular ablation. Group 2, the comparison group, included the material from the patients with high PH (24 histological samples), which was not ablated. The third, control group included the material from the patients without PH, whose cause of death was not cardiovascular pathology. These samples (35 histological samples) were not exposed to radiofrequency, either.

Of all the samples of the PA wall, two sections were formed, 6 fields were isolated in each of them for visual analysis. Further, as the optical density was determined, up to ten repeated calculations were additionally performed for each field of view. After radiofrequency exposure, the samples of the group 1 were fixed in a 10% solution of buffered neutral formalin, the volume of which was 10 or more times the volume of the material itself. The samples were treated for 36 h at room temperature.

**Figure 2.**
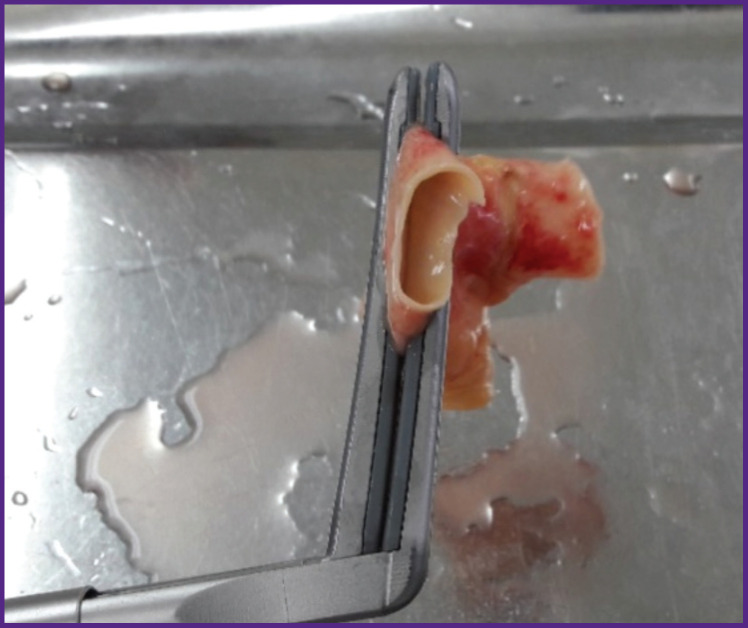
Ablation of the pulmonary artery with an ablation clamp

Circularity of radiofrequency exposure was achieved due to tight and plane fixation of the test samples in the jaws of the ablation clamp using the proprietary technique [18]. The walls of the PA vessel were exposed to controlled radiofrequency with the electrodes built into the jaws, supplied with the energy delivered from an automatically programmable generator (Figure 2).

During the ablation procedure, continuous hardware monitoring of the degree of tissue conductance was carried out with automatic calculation of the impedance and its imaging in a dynamic graphical form with the estimated margins of the expected level of transmural tissue damage.

**Figure 3.**
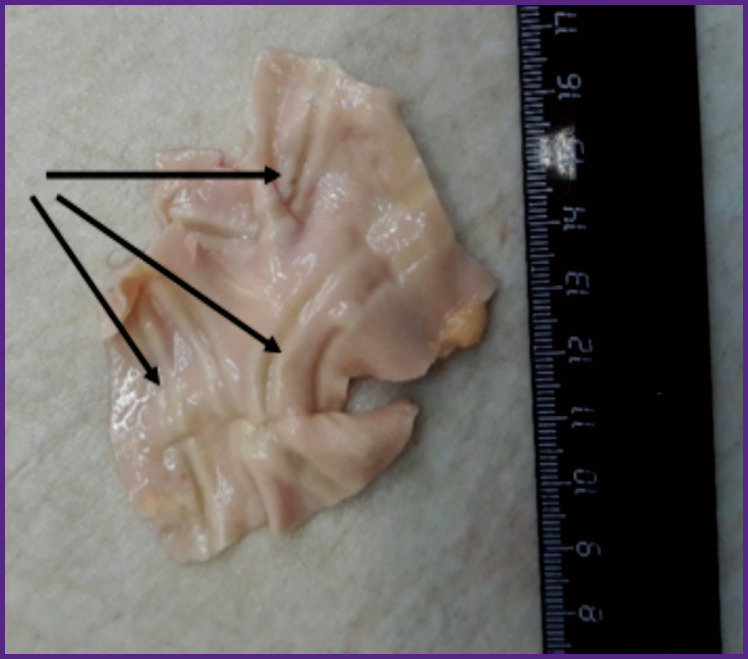
Ablation lines (*shown by arrows*) formed on the outer surface of the samples after radiofrequency exposure

Two ablation procedures were performed on the pulmonary trunk and on the orifices of each PA with the formation of 6 ablation lines, macroscopically visualized as imprints, including those from the mechanical impact, on the outer surface of the PA vessel samples (Figure 3).

**Figure 4.**
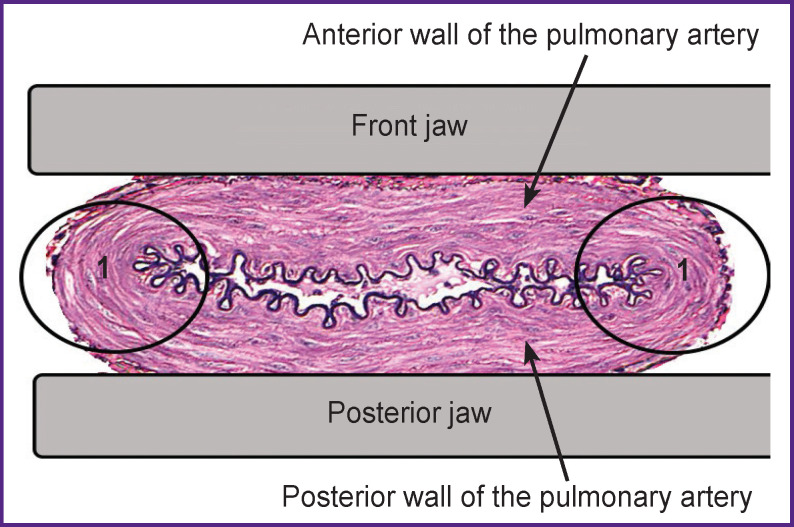
Scheme of fixation of the walls of the pulmonary artery with the jaws at their transverse clamping *1* — “Marginal zones”

During the ablation process, the fixation of the PA walls in the plane parallel jaws of the ablation clamp leads to tissue spreading due to transverse clamping, which results in the formation of conditional “marginal zones” (Figure 4), in which the applied mechanical pressure also produces a “tearing” effect.

Taking into account the unevenness of transverse mechanical compression, the material from the group 1 (depending on the tissue area exposed to ablation) was additionally divided into two subgroups. Subgroup 1A included areas of the central zone of the PA transverse section (108 histological samples), subgroup 1B included areas of the “marginal zone” (99 histological samples).

Light microscopy and methods of staining the formed complexes were used to determine the signs indicating the effect of performed radiofrequency. The typical general pathological processes were recorded by hematoxylin and eosin staining. The transmurality of exposure was determined by staining according to Van Gieson. Impregnation with silver salts (according to Ramón y Cajal) was used to visualize the reticular fibers, trunks and endings of the peripheral nerve fibers.

The degree of thermal ablation effect was assessed using a semi-quantitative analysis (per ten fields of view) of all the identified pathological processes. Mathematical assessment was performed using the computer morphometry of the sections obtained with an Olympus SP-350 camera in the optics of a Leica CME microscope (Leica Microsystems, Germany).

### Statistical data processing

The results were statistically evaluated using the SPSS Statistics 26.0 software. The mean and standard deviation (M±σ) was used to describe the quantitative data. The Student’s t-test was used to perform statistical hypothesis testing in the presence of a normal distribution of the initial data, and the Mann–Whitney U-rank test was used in the case of unequal variances. The Kruskal–Wallis test for quantitative and rank data and Pearson’s χ^2^ test for qualitative data were used to compare the groups. The differences were considered statistically significant at p<0.05.

## Results

The study of the histological samples of all the groups at the light-optical level allowed determining the qualitative signs of the radiofrequency effect.

**Figure 5.**
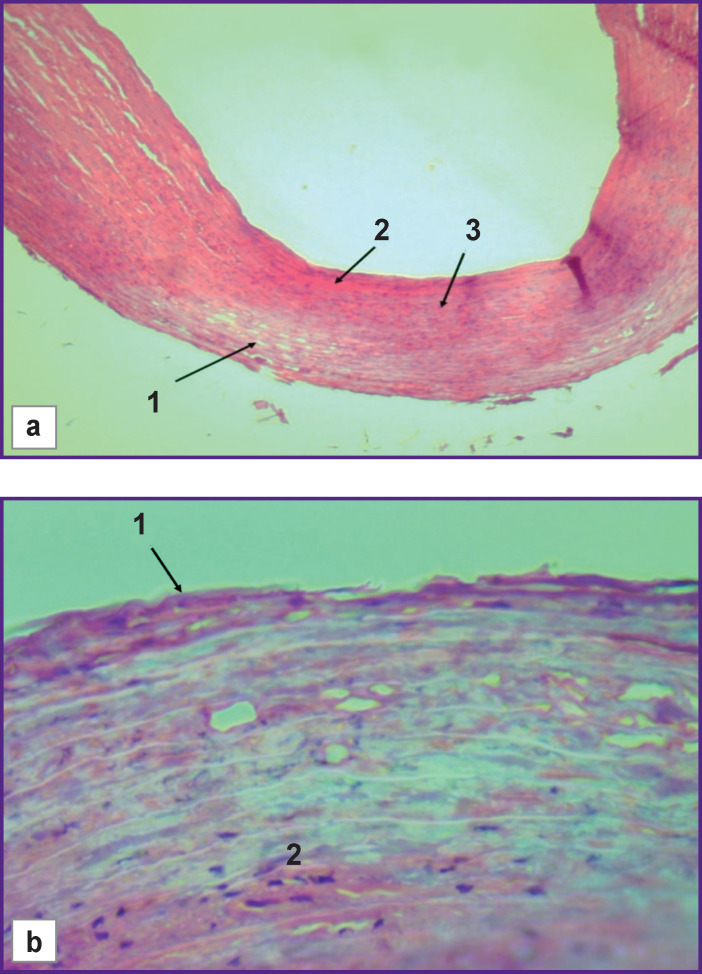
Transverse section of the pulmonary artery wall in the area of radiofrequency exposure (staining with hematoxylin and eosin): (a) “marginal zone” (subgroup 1B): *1* — thinning of elastic fibers under the adventitia; *2* — necrosis in the subendothelial layer — fibrinoid necrosis; *3* — metachromasia foci; ×40; (b) central zone (subgroup 1A): *1* — superficial mechanical desquamation in the adventitial layer; *2* — phenomena of karyorrhexis and karyolysis (fibrinoid necrosis); ×400

Thus, when staining with hematoxylin and eosin from the side of the PA adventitia membrane, the fields of elastic fibers with the signs of pronounced disorganization in the form of thinning and rarefaction were determined, which indicated the destruction of dense intercellular associations in the media of the vessel (Figure 5 (a)). In the presence of these alterations in the structure of fibroblasts and smooth myocytes, the phenomena of karyorrhexis and karyolysis were observed (Figure 5 (b)). Moreover, the foci of metachromasia were detected in some areas of the subendothelial layer among the areas of fibrinoid necrosis (see Figure 5 (a)). This may indicate a lower radiofrequency energy distribution in this layer. In general, deep transmural disorganization of the tissues of the PA wall was observed after radiofrequency ablation.

**Figure 6.**
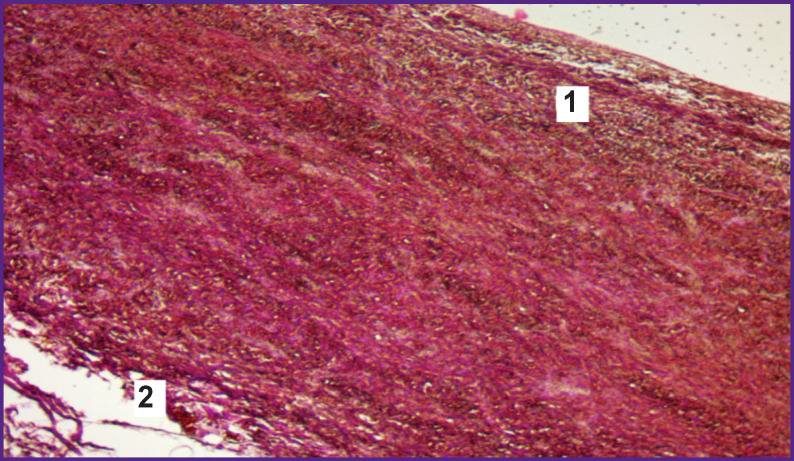
“Marginal zone” (subgroup 1B) of the transverse section of the ablated area (staining according to Van Gieson; ×10): *1* — areas of fibrinoid necrosis under the adventitial layer; *2* — mechanical crushing of tissue in the subendothelial layer

The tissue sections stained according to Van Gieson (Figure 6), as well as those stained with hematoxylin and eosin, had areas of fibrinoid necrosis, located directly in the area adjoining the electrode surface of the jaws of the ablation clamp to the adventitia layer, which is due to the action of the current in the radiofrequency range. In the subendothelial layer of the “marginal zones” of the vessel, areas of tissue crush were observed due to the rupturing effect of the applied mechanical compression.

**Figure 7.**
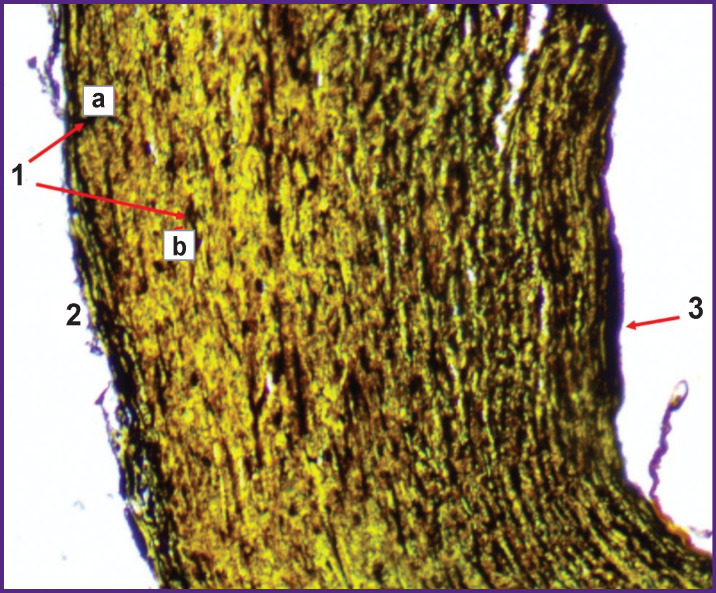
“Marginal zone” (subgroup 1B) of the transverse section of the ablated area (impregnation with silver salts; ×100): *1* — zones of staining nerve endings (a) and reticulin fibers (b) with metallic silver; *2* — adventitia; *3* — subendothelial layer with silver deposits

The impregnation of samples with silver salts made it possible to determine pathological changes in argyrophilic fibers (Figure 7).

The decreased density of the nerve tissue in the outer layer adjacent to the electrodes demonstrates a positive effect of radiofrequency denervation of sympathetic nerve fibers after performing PADN.

**Figure 8.**
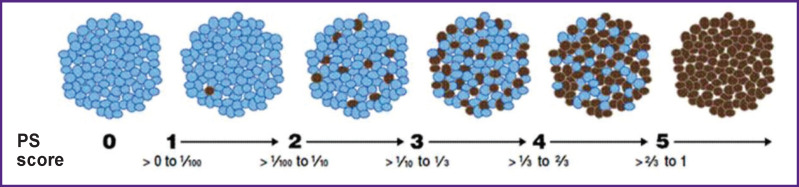
Scoring system for the color intensity according to Allred [19]

To assess the radiofrequency exposure level, the scoring method of semi-quantitative analysis of pathological processes (assessment of the color intensity according to Allred) was used (Figure 8) [19].

To conduct the intergroup analysis of the histological results, a table was formed with scoring for each attribute for all the study groups (Table 1). The pathological signs in the form of fibrinoid necrosis, metachromasia, and pronounced disintegration of collagen fibers were most often observed in subgroup 1B, which is explained by the formation of tension zones as a result of the almost complete bending of the PA wall duplication under compression of the jaws of the ablation device. In the comparison and control groups, no signs of significant pathological disorganization of PA tissues were observed, and the density of nerve tissue structures impregnated with silver salts (reticular fibers, trunks and endings of peripheral nerve fibers) was the highest among all the samples of these two groups.

**Table 1:** Semi-quantitative analysis of the pathological signs of the thermal ablation effect (points)

Pathological sign	Ablation group (n=207)		Comparison group (n=24)	Control group (n=35)
	Subgroup 1A (central zone, n=108)	Subgroup 1B (“marginal zone”, n=99)		
Fibrinoid necrosis	3	4	1	0
Metachromasia	2	4	0	0
Dissociation of collagen media fibers	3	5	0	0
Reticular fibers, trunks and endings of peripheral nerve fibers	3	2	5	4

In all the samples after thermal ablation, uneven staining of the PA wall structures with silver salts was observed, its lower distribution being closer to its adventitial layer, which is likely due to the development of pathological changes in the structures of both the reticular and nerve fibers after radiofrequency exposure.

To determine the quantitative representation of the density and level of distribution of the radiofrequency effect, the detected pathological signs were assessed morphometrically (Table 2).

**Table 2:** Assessment of density and distribution level of the determined pathological signs

Pathological sign	Ablation group (n=207)		Comparison group (n=24)	Control group (n=35)
	(central Subgroup zone, 1n=A) 108)	(“marginal Subgroup zone”, 1B n=99)		
The mean optical density of the PA wall (rel. units), M±σ	0.17±0.02*	0.19±0.03*	0.88±0.13	0.33±0.05
Mean specific area of argentophilic fibers (%), Р±σ_Р_	56.34±3.10*	57.75±2.70*	73.10±2.10	65.81±1.80
Mean specific area of connective tissue disorganization (%), Р±σ_Р_	30.0±4.20	43.20±1.90	—	—

* Statistically significant differences with the comparison and control groups (p<0.05).

To compare a degree of disorganization of the fibrous structures of the vessel media, their optical density, equal to the decimal logarithm of the difference in light transmission through the object, was calculated. The mean optical density of the samples of the ablation group was found to be statistically significantly lower than in the comparison and control groups (p<0.001), despite the differences in the degree of disorganization of the fibrous structures in the connective tissue of the middle PA layer among the samples of the subgroups (1A — 0.17±0.02; 1B — 0.19±0.03). When calculating the specific area of connective tissue disintegration, its mean value in the samples of the central zone (subgroup 1A) was 30.0%, and in the samples of the “marginal zone” (subgroup 1B), it was 43.2% of the tissue area in the field of view. The development of such pathological signs was caused by the pronounced mechanical compression of tissues by the jaws of the ablation clamp, with a greater severity in the “marginal zones” of the sections due to the bending of the PA walls and practically bursting of the fibrous structures of the wall.

The mean area of argentophilic fibers in the ablation subgroups did not differ statistically significantly. At the same time, there was a difference in these values compared to the untreated tissues, namely, in a decrease (by 16%) in the content of argentophilic fibrous structures (p˂0.05). The highest concentration of argentophilic structures was observed in the comparison group, the samples of which, as in the ablation group, were taken from patients with high PH, but they were not exposed to radiofrequency. Disorganization of the fibers of the middle PA layer in the groups with performed ablation was constant and weakly variable. The silver salt deposition was noted to be considerably lower in the adventitia layer and structures of the PA wall, close to it and exposed to radiofrequency. In this case, the mechanical compression by the jaws did not affect the distribution of impregnation.

Thus, based on the qualitative and quantitative analysis of the pathological signs in the samples of all the groups, the distinctive features of the thermal ablation effect as various degrees of severity of connective tissue disorganization, manifested to a greater extent by the presence of fibrinoid necrosis in the subadventitious layers and, to a lesser extent, by the development of mucoid swelling in the subendothelial layer. In the “marginal zones”, due to a more pronounced mechanical compression, the described features are found in a larger area, have a more complicated nature, and are distributed up to the intima.

Taking into account the circularity of the distribution of sympathetic nerve plexuses in the wall adventitia in the region of the trunk and PA bifurcation, an even distribution of thermal ablation energy is required along the entire circumference of the treated adventitia to perform high-quality denervation, without affecting the deep layers with vasoconstrictor sympathetic nerve fibers located in them.

It should be remembered that, like other large pulmonary vessels, the trunk and bifurcation region is a strong reflexogenic zone involved in neural reflex regulation of the pulmonary circulation vessels [20]. The baroreceptors distributed in the intimate layer of the main branches of the PA are responsible for this regulation. The baroreceptor activation with an increase in intravascular pressure in the PA due to a decrease in the frequency of heart contractions and vasoconstriction in the systemic circulation leads to a decrease in pressure in the systemic circle and the deposition of blood in the body, which ultimately reduces venous return to the heart and lung blood vessels (Parin’s reflex). This reflex is dramatically important in unloading the vessels of the pulmonary circulation, protecting the right ventricle from overload, and preventing the decompensation of the pulmonary circulation, including the development of acute pulmonary edema [10, 13, 15]. Therefore, damage to the intimate layer of arterial vessels will have a negative impact on the work of one of the compensatory mechanisms activated in patients with the PA development.

## Conclusion

The results of histological examination showed the feasibility of radiofrequency ablation of the pulmonary arteries in patients with high-grade secondary pulmonary hypertension. Radiofrequency denervation leads to the destruction of the sympathetic ganglia in the adventitial layer of the pulmonary arteries responsible for the spasm of the precapillary bed of the pulmonary circulation, which contributes to vasodilation, an increase in the capacity of the vascular bed, and, as a consequence, a reduction in pulmonary hypertension.
